# Do hospitalized children with obesity lose more weight than eutrophic ones?

**DOI:** 10.1590/1806-9282.20251923

**Published:** 2026-10-23

**Authors:** Dalila Nunes Alcantara, Karina Guimarães de Angelis, Adriana Sañudo, Maria Teresa Bechere Fernandes

**Affiliations:** 1 Universidade de São Paulo, Faculdade de Medicina, Department of Pediatrics – São Paulo (SP), Brazil.; 2 Universidade Federal de São Paulo, Department of Preventive Medicine – São Paulo (SP), Brazil.

**Keywords:** Teenagers, Children, Hospital, Hospitalization, Overweight, Weight loss.

## Abstract

**BACKGROUND:**

The clinical course of hospitalized children with overweight has been scarcely investigated. Previous studies suggest that these children experience longer hospital stays and greater weight loss than their normal-weight peers, potentially compromising lean body mass and immune function.

**OBJECTIVE:**

The aim of this study was to investigate the association between nutritional status at hospital admission and hospital length of stay, and to compare weight loss trajectories between children with overweight and those with normal weight.

**METHODS:**

This prospective study included 125 hospitalized children and adolescents aged 2–14 years. Anthropometric measurements and sociodemographic and clinical data were collected. Associations with hospital length of stay were assessed using multiple Poisson regression, and weight loss trajectories were analyzed using analysis of covariance.

**RESULTS:**

Of the 125 participants, 56.8% were male, and 29.6% had overweight. Asthma was the most common reason for hospitalization (25.6%). Children with overweight had a 25% longer hospital stay than those with normal weight. Among children with overweight, each additional day of hospitalization was associated with a mean weight loss of 0.20 kg (95%CI 0.14–0.26) and a 0.04-unit decrease in body mass index-for-age z-score (95%CI 0.01–0.06).

**CONCLUSION:**

Overweight at hospital admission was associated with a longer hospital stay and progressive weight loss during hospitalization.

## INTRODUCTION

Pediatric overweight and obesity have reached alarming levels worldwide, including in Brazil. A nationwide study including more than five million Brazilian children aged 3–10 years reported an increase in the prevalence of excess weight of approximately 3% among those aged 5–10 years and 1% among those aged 3–4 years between 2001 and 2014^1^.

Although hospital malnutrition has been extensively investigated, the impact of excess weight on hospitalization outcomes remains poorly understood. In Brazil, the prevalence of overweight among hospitalized children ranges from 8.9 to 25.2%^2,3^, while international studies have reported prevalences ranging from 17.3 to 30%^4, 5, 6^.

A systematic review published in 2013 identified childhood obesity as a risk factor for increased mortality in severe illnesses^7^. In addition, children with obesity are more likely to require hospitalization for orthopedic, respiratory, surgical, gastrointestinal, and genitourinary conditions, resulting in increased healthcare utilization and hospital costs^4,8^.

Excess weight has also been consistently associated with asthma. A 2019 meta-analysis including 73,252 children reported a higher risk of asthma among children with overweight (OR 1.30; 95%CI 1.23–1.39)^9^, and a subsequent meta-analysis confirmed this association (OR 1.50; 95%CI 1.30–1.70)^10^. Likewise, a retrospective cohort study found that children with overweight were more likely to be hospitalized because of asthma (OR 1.60; 95%CI 1.00–2.60)^11^.

Potential mechanisms include obesity-related immune dysregulation and chronic low-grade inflammation, which may contribute to poorer clinical outcomes and prolonged hospital stays^2,12,13^.

Given the limited evidence on the clinical course of hospitalized children with overweight, this study aimed to investigate the association between nutritional status at hospital admission and hospital length of stay and to compare weight loss trajectories during hospitalization between children with overweight and those with normal weight.

## METHODS

### Study design and participants

This prospective cohort study included 125 children and adolescents aged 2–14 years who were admitted to the University Hospital of the Universidade de São Paulo, Brazil, between 2014 and 2017. Only the first hospitalization during the study period was considered for inclusion. Children younger than 2 years of age or with chronic diseases were excluded.

The sample size was estimated using G*Power version 3.1, assuming mean hospital stays of 5.5 and 3.7 days for children with overweight and normal weight, respectively, a common standard deviation (SD) of 2.6 days, a two-sided α of 0.05, and 80% statistical power, resulting in a minimum sample size of 33 participants per group^2^. To ensure adequate statistical power, 88 children with normal weight and 37 with overweight were included.

### Data collection and variables

Sociodemographic information, including child and maternal characteristics and family income, was obtained through structured interviews. Family income was expressed as multiples of the Brazilian minimum wage. Clinical data, including the primary reason for hospitalization, antibiotic use, oxygen therapy, and admission to the intensive care unit (ICU), were obtained from medical records.

Anthropometric measurements were performed by trained researchers within 24 h of hospital admission. Body weight was measured daily using a calibrated FILIZOLA^®^ digital scale, and height was measured using an ALTURAEXATA^®^ stadiometer according to standardized procedures^14,15^. Waist circumference was measured at the midpoint between the lowest rib and the iliac crest.

Nutritional status was classified according to the World Health Organization (WHO) growth standards^16^ using WHO Anthro version 3.2.2 and WHO AnthroPlus version 1.0.4 to calculate body mass index (BMI)-for-age (BAZ) and height-for-age (HAZ) z-scores. The overweight group comprised children classified as at risk of overweight, overweight, or obese. Central adiposity was assessed using the waist-to-height ratio, with values ≥0.50 indicating abdominal adiposity^17^.

### Statistical analysis

Categorical variables are presented as frequencies and continuous variables as mean (SD) or median (interquartile range), as appropriate. Because hospital length of stay was not normally distributed, the variable was log-transformed and is presented as the geometric mean with a 95%CI.

Factors associated with hospital length of stay were evaluated using Poisson regression models. Variables with p<0.20 in the univariate analyses were included in the multivariable model. Results are presented as relative risks with corresponding 95%CI.

Pearson correlation coefficients were used to examine the associations between hospital length of stay and changes in body weight (Δweight) and BMI-for-age z-score (ΔBAZ). Delta values were calculated as the difference between discharge and admission measurements, with negative values indicating weight loss.

Changes in body weight and ΔBAZ were further analyzed using analysis of covariance (ANCOVA), with nutritional status as the fixed factor and hospital length of stay as a covariate. Interaction terms were included to evaluate whether the association between hospital length of stay and anthropometric changes differed according to nutritional status. Statistical significance was defined as a two-sided p<0.05. All analyses were performed using STATA/MP version 18.0 (StataCorp LLC, College Station, TX, USA).

### Ethical considerations

The study was approved by the Research Ethics Committee of the Faculty of Medicine and the University Hospital of the Universidade de São Paulo (CAAE: 27944414.6.0000.0065). Written informed consent was obtained from the parents or legal guardians of all participants before enrollment.

## RESULTS

The study included 125 hospitalized children and adolescents with a mean age of 7.4 years, of whom 56.8% were male. Mothers had a mean of 9.6 years of schooling, and the median family income was 2.1 times the Brazilian minimum wage. At hospital admission, 70.4% of participants had normal weight and 29.6% had overweight. The mean WHtR was 0.50, indicating central adiposity in a substantial proportion of the sample. The most common reasons for hospitalization were asthma (25.6%), cellulitis (14.4%), and appendicitis (12.8%). The median hospital length of stay was 4 days. Overall, 68.0% of patients received antibiotics, 22.6% received oxygen therapy, and 6.5% required admission to the ICU (Table 1).

**Table 1.** Description of the sample according to sociodemographic and clinical data (n=125).

	Mean (SD)/median (P25–P75) Minimum–maximum	n (%)
Age (years)	7.4 (3.4)	
	2.4–14.9	
Sex		
Female		54 (43.2)
Male		71 (56.8)
Maternal age (years)	32.6 (7.5)	
	18.0–52.0	
Education (years)	9.6 (3.0)	
	1–16	
Income (minimum wage)	2.1 (1.4–3.8)	
	0.4–12.7	
Nutritional status		
Eutrophy		88 (70.4)
Risk of overweight		4 (3.2)
Overweight		13 (10.4)
Obesity		20 (16.0)
HAZ (z-escore)	0.2 (1.3)	
	-2.6–3.2	
BAZ (z-escore)	0.5 (1.3)	
	-2.0–3.6	
Waist (cm)	58.5 (52.0–66.0)	
	43.0–100.0	
Waist-to-height ratio	0.5 (0.1)	
	0.4–0.7	
Diagnosis at the time of hospitalization		
Abscess		3 (2.4)
Appendicitis		16 (12.8)
Acute appendicitis with abscess		7 (5.6)
Septic arthritis		2 (1.6)
Asthma		32 (25.6)
Asthma and pneumonia		11 (8.8)
Acute bronchiolitis		2 (1.6)
Cellulitis		18 (14.4)
Other infectious diseases		13 (10.4)
Diabetes mellitus–insulin-dependent		5 (4.0)
Fracture		3 (2.4)
Diseases of the nervous system		3 (2.4)
Others		10 (8.0)
Length of hospitalization (days)	4 (2–7)	
	1–36	
Use of antibiotics		
No		40 (32.3)
Yes		84 (67.7)
Use of oxygen		
No		96 (77.4)
Yes		28 (22.6)
ICU		
No		115 (93.5)
Yes		8 (6.5)

SD: standard deviation; HAZ: height-for-age z-score; BAZ: body mass index-for-age z-score; ICU: intensive care unit.

In the multivariable Poisson regression analysis, overweight status, antibiotic use, and ICU admission were independently associated with longer hospital stays (p<0.05), whereas oxygen therapy was associated with a shorter length of stay (p<0.001) (Table 2). Children with overweight experienced greater reductions in body weight (Δweight=-0.09±0.36 kg) and BMI-for-age z-score (ΔBAZ=-0.09±0.36) than children with normal weight (Δweight=-0.03±0.43 kg).

**Table 2.** Multiple Poisson regression model for length of hospitalization.

Multiple Poisson regression*	RR_adjusted_	95%CI	p
Nutritional status			
Weight excess×eutrophy	1.25	1.02–1.55	0.036
Use of antibiotics (yes×no)	1.70	1.24–2.34	0.001
Use of oxygen (yes×no)	0.54	0.40–0.72	<0.001
ICU (yes×no)	2.21	1.52–3.22	<0.001

*Model adjusted for child’s age, hospitalization diagnosis, maternal education, and income. CI: confidence interval; ICU: intensive care unit.

Among children with normal weight, hospital length of stay was not significantly correlated with changes in body weight or BMI-for-age z-score. In contrast, among children with overweight, longer hospital stays were significantly associated with greater reductions in both body weight (r=-0.656) and BMI-for-age z-score (r=-0.540). ANCOVA confirmed a significant interaction between nutritional status and hospital length of stay for both Δweight (interaction β=-0.23) and ΔBAZ (interaction β=-0.05; both p<0.01). Specifically, among children with overweight, each additional day of hospitalization was associated with an average weight loss of 0.20 kg and a 0.04-unit decrease in BMI-for-age z-score (Figure 1).

**Figure 1:**
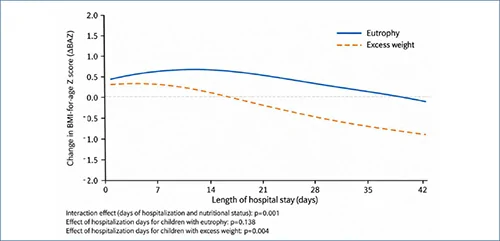
Interaction effect—days of hospitalization and nutritional status.

## DISCUSSION

This study showed that children with overweight had a 25% longer hospital stay than children with normal weight and experienced progressive, time-dependent weight loss during hospitalization.

Between 2014 and 2017, 29.6% of hospitalized children in our cohort had overweight, corresponding to an average annual prevalence of 7.4%. This prevalence was comparable to that reported in a previous study conducted at the same institution 5 years earlier (8.9% annually)^2^, but lower than the 25.2% observed at a tertiary referral hospital in Recife^3^. Differences in age eligibility, particularly the inclusion of infants younger than 1 year in the Recife study, may partly explain this discrepancy. International studies have reported prevalences ranging from 17.3 to 30% among hospitalized children and adolescents^4,6^. It is important to emphasize that these estimates represent the prevalence of overweight among hospitalized patients rather than in the general pediatric population. Consequently, they reflect a subgroup of children requiring hospital care and should not be directly compared with population-based prevalence estimates.

Asthma, cellulitis, and appendicitis were the leading causes of hospitalization in the present study. Asthma was also the most frequent diagnosis in a previous study conducted at our institution^2^, and respiratory diseases remain one of the leading causes of pediatric hospitalization worldwide^6,18^.

Overweight, antibiotic use, and ICU admission were independently associated with longer hospital stays. Antibiotic therapy and ICU admission are likely markers of greater disease severity and clinical complexity. Conversely, oxygen therapy was associated with a shorter hospital stay, although this finding may reflect differences in clinical management or patient characteristics rather than a direct therapeutic effect.

Although overweight remained independently associated with prolonged hospitalization, hospital length of stay in pediatric patients should be viewed as a multidimensional outcome rather than a direct consequence of disease severity or nutritional status alone. Length of stay reflects the interaction of disease severity, recovery trajectory, care complexity, and healthcare delivery processes. Consistent with this perspective, a recent study demonstrated that nursing care demands independently contributed to prolonged hospitalization among children and adolescents, even after adjustment for diagnosis-related group severity and other clinical characteristics^19^. Therefore, the longer hospital stays observed among children with overweight may also reflect greater care complexity and evolving supportive care needs during hospitalization, rather than exclusively the biological effects of excess adiposity.

Our findings are consistent with previous pediatric studies showing that overweight and obesity are associated with prolonged hospitalization across a range of clinical settings, including respiratory diseases, surgical admissions, and COVID-19^4,6,20,21,22^. Together, these findings support the hypothesis that excess adiposity is associated with less favorable hospital outcomes.

The most important finding of the present study was the progressive, time-dependent weight loss observed among children with overweight during hospitalization. Each additional day of hospitalization was associated with an average reduction of 0.20 kg in body weight and a 0.04-unit decrease in BMI-for-age z-score. No similar pattern was observed among children with normal weight. To our knowledge, few pediatric studies have examined longitudinal changes in anthropometric indicators throughout hospitalization. A previous study conducted at our institution similarly reported greater weight z-score loss among hospitalized children with overweight than among those with normal weight^2^.

Several mechanisms may contribute to these findings. During acute illness, surgery, or trauma, children with overweight may develop a hypercatabolic response characterized by disproportionate loss of lean body mass relative to fat mass^23^. This metabolic response may impair immune function and contribute to poorer clinical outcomes in children with obesity, particularly those hospitalized with respiratory conditions such as asthma^10, 11, 12,24^. Nevertheless, these mechanisms remain speculative in the context of the present study and warrant further investigation.

The observed association between prolonged hospitalization and progressive weight loss should also be interpreted with caution. The overweight group included children ranging from at risk of overweight to obesity, representing a clinically heterogeneous population. Moreover, because body composition was not assessed, it was not possible to determine whether the observed weight loss reflected reductions in fat mass, lean mass, or body water. Future prospective studies incorporating body composition assessment and metabolic biomarkers are needed to better characterize the mechanisms underlying weight loss during hospitalization in children with overweight.

This study has some limitations. First, the sample size was relatively small (n=125) compared with the total number of pediatric hospitalizations during the study period (n=2,745). However, comparisons between included and non-included patients suggested that selection bias was minimal. Second, this was a single-center study, which may limit the generalizability of the findings to other healthcare settings.

## CONCLUSION

Overweight at hospital admission was independently associated with a longer hospital stay and progressive, time-dependent weight loss during hospitalization. These findings suggest that children with overweight may represent a subgroup at increased nutritional vulnerability during hospitalization and highlight the importance of careful nutritional monitoring throughout the hospital stay. Further prospective studies incorporating body composition and metabolic assessments are needed to clarify the mechanisms underlying these findings and their potential implications for clinical outcomes.

## Data Availability

The datasets generated and/or analyzed during the current study are available from the corresponding author upon reasonable request.
